# THE ACHIEVE RANDOMIZED TRIAL: EFFECT OF HEARING LOSS INTERVENTION ON COGNITIVE DECLINE IN OLDER ADULTS

**DOI:** 10.1093/geroni/igad104.0295

**Published:** 2023-12-21

**Authors:** Jennifer Deal

**Affiliations:** Johns Hopkins University, Baltimore, Maryland, United States

## Abstract

Hearing loss in older adults is linked to accelerated cognitive decline and incident dementia in population-based observational studies; whether intervention can reduce cognitive decline is unknown. The multi-center NIH-funded ACHIEVE randomized controlled trial is testing efficacy of a best-practices hearing intervention (amplification and rehabilitation) versus a successful aging health education control intervention, on 3-year cognitive decline in 977 dementia-free adults aged 70-84 years with untreated mild-to-moderate hearing loss. This presentation will describe results for the primary outcome of 3-year cognitive decline, measured using a 10-item neurocognitive battery summarized using latent variable methods. Secondary outcomes include (1) domain-specific cognitive declines in memory, language and executive function and (2) time until cognitive impairment defined as a composite of adjudicated dementia or mild cognitive impairment diagnosis, a 3-point drop in the 30-item Mini-Mental State Exam (MMSE) administered in-person, or a 3-point drop in a factor score derived from the 10-item MMSE orientation subscale and 11-item Blessed scale administered over the phone and rescaled to be equivalent to the 30-item MMSE. The trial is ongoing with data closeout March/April 2023; data will be analyzed per the ACHIEVE Coordinating Center statistical analysis plan, approved by the NIA and ACHIEVE DSMB. Although preliminary results are not possible to include here to protect trial integrity, results will be available to present for GSA. ACHIEVE is the first large-scale randomized study to determine if hearing loss intervention reduces cognitive decline. Whether findings are positive or null, this study will have substantial clinical and public health impact.

